# *Klebsiella pneumoniae* capsular polysaccharide: Mechanism in regulation of synthesis, virulence, and pathogenicity

**DOI:** 10.1080/21505594.2024.2439509

**Published:** 2024-12-13

**Authors:** Li Xu, Jiayang Li, Wenqi Wu, Xiuwen Wu, Jianan Ren

**Affiliations:** a Research Institute of General Surgery, Jinling Hospital, the Affiliated Hospital of Medical School, Nanjing Medical University, Nanjing, China; b Research Institute of General Surgery, Jinling Hospital, the Affiliated Hospital of Medical School, Nanjing University, Nanjing, China

**Keywords:** *Klebsiella pneumoniae*, capsular polysaccharide, structure and typing, virulence, pathogenic mechanisms, immune evasion

## Abstract

Hypervirulent *Klebsiella pneumoniae* exhibits strong pathogenicity and can cause severe invasive infections but is historically recognized as antibiotic-susceptible. In recent years, the escalating global prevalence of antibiotic-resistant hypervirulent *K. pneumoniae* has raised substantial concerns and created an urgent demand for effective treatment options. Capsular polysaccharide (CPS) is one of the main virulence determinants contributing to the hypervirulent phenotype. The structure of CPS varies widely among strains, and both the structure and composition of CPS can influence the virulence of *K. pneumoniae*. CPS possesses various immune evasion mechanisms that promote the survival of *K. pneumoniae*, as well as its colonization and dissemination. Given the proven viability of therapies that target the capsule, improving our understanding of the CPS structure is critical to effectively directing treatment strategies. In this review, the structure and typing of CPS are addressed as well as genes related to synthesis and regulation, relationships with virulence, and pathogenic mechanisms. We aim to provide a reference for research on the pathogenesis of *K. pneumoniae*.

## Introduction

*Klebsiella pneumoniae*, a well-known gram-negative iatrogenic bacterium, is a major opportunistic pathogen that may cause several health care-associated infections, such as pyogenic liver abscess, bacteremia, and urinary tract infection [1,2]. In China, *K. pneumoniae* has become the second most prevalent pathogen involved in clinical infections. *K. pneumoniae* can be classified into two main types: classical *K. pneumoniae* (cKP) and hypervirulent *K. pneumoniae* (hvKP). cKP commonly causes infections in patients with comorbidities or inherent vulnerabilities and is a major contributor to nosocomial infections. These infections often exhibit antibiotic resistance, particularly to carbapenems, leading to limited treatment options [1]. HvKP can induce severe and life-threatening invasive community-acquired infections in community-dwelling healthy individuals, generally showing limited sensitivity to most antibiotics but possessing high virulence [3]. Currently, there is notable concern about the rise of multidrug-resistant hypervirulent *K. pneumoniae* (MDR-hvKP), which is simultaneously highly pathogenic and resistant to many antibiotics. This phenomenon is mainly caused by the horizontal transfer of drug resistance or virulence genes via plasmids [4,5].

*K. pneumoniae* uses a range of virulence factors to ensure its survival and promote disease development; these factors include capsule, lipopolysaccharide (LPS), siderophore, types I and III fimbriae, outer membrane proteins, and the type VI secretion system [6]. The hvKp strains typically produce a very thick capsule on their surface and often have a hypermucoviscous phenotype [7]. The overproduction of capsule plays a dominant role in virulence and pathogenicity by providing protection for the bacterium against attacks from the complement system and phagocytosis. The *K. pneumoniae* capsule is primarily composed of polysaccharides, and the structural characteristics of these polysaccharides are pivotal in determining the capsule properties. The relationship between capsular polysaccharide (CPS) and virulence has been studied in various mouse models of *K. pneumoniae* infection, including pneumonia, peritoneal infection, and urinary tract infection [8,9]. Therefore, this review is aimed at clarifying the function of CPS in *K. pneumoniae* to contribute to the advancement of treatment strategies by examining its structural characteristics, classification, regulation mechanisms, and pathogenic mechanisms.

## Structure and typing of capsular polysaccharide (CPS)

The capsule is a loosely arranged mucoid layer located on the exterior of the *K. pneumoniae* bacterium, primarily composed of a repeating glycan polymer that effectively protects the organism against various immune responses and adapts it to different environments [10]. As a major virulence determinant of *K. pneumoniae*, CPS is the primary component of the capsule and has a crucial function in limiting the immune response. Adaptation of *K. pneumoniae* to novel environments is facilitated by the presence of the capsule, which is achieved by fine-tuning expression of *cps* and altering those traits associated with virulence [11]. Encapsulated *K. pneumoniae* adapts to a novel environment by increasing its viscosity or by adopting a hypermucoviscous phenotype whereas non-capsulated *K. pneumoniae* increases its production of surface polysaccharide and biofilm formation via different mechanisms.

Capsular antigens, also known as K-antigens, are composed of three to six monosaccharides in their main chain and branch, including mannose, glucose, galactose, fucose, and rhamnose. The presence of glucuronic acid and galacturonic acid can render K-antigens anionic. Pyruvate, O-acetyl, and O-formyl are involved in the modification of polysaccharides, which leads to diversity among capsules and mediates the immunogenicity and virulence levels of *K. pneumoniae* [12]. In K1 CPS, pyruvation and O-acetylation increase the production of pro-inflammatory cytokines, including interleukin-6 (IL-6) [13]. It has been demonstrated that in K57 CPS, acetylation reduces bacterial serum resistance and increases adhesion to intestinal epithelial cells, thereby contributing to pathogenicity [14]. Further investigation is required to understand the pathogenic mechanisms of capsule modification in *K. pneumoniae* with different K types.

Traditional serotyping methods classify *K. pneumoniae* into at least 79 chemically distinct serotypes according to variations in the capsule constituents and structure [15,16]. The diverse sugar composition of the main and side chains of 79 *K. pneumoniae* K-antigens has been previously summarized [17]. However, owing to frequent loss and acquisition of capsule loci by horizontal gene transfer [18], up to 70% of *K. pneumoniae* are either capable of producing a novel capsule or are incapable of expressing any capsule, meaning that these bacteria are not typeable using serotyping methods. Recently, new typing methods have been reported owing to advancements in molecular techniques and sequencing technologies. These are known as KL types [19] and are based on the sequence of conserved *wzi*, *wzy*, or *wzc* of the *cps* locus, or on whole genome data, which is more precise [20,21]. As many as 186 KL types have been identified using comparative genomics, and the KL1–KL81 locus types correspond directly to the K1–K81. As increasing numbers of *K. pneumoniae* genomes become available, additional novel K loci are expected to be discovered in the future [22–24].

Different serotypes show great variations in virulence characteristics, with hvKP commonly found in the specific serotypes K1, K2, K16, K28, K54, K57, and K63 [25]. K1, K2, K5, K20, K54, and K57 serotypes are often linked to pyogenic liver abscesses whereas K2, K1, K57, K5, K20, and K54 serotypes are commonly linked to meningitis [14,26]. It has been demonstrated that K1, K2, K16, and K20 hvKP exhibit very high levels of virulence in a mouse model of intraperitoneal infection [9]. Thus, both clinical symptoms and laboratory tests confirm that certain serotypes are reliable indicators of high virulence levels in *K. pneumoniae*.

Notably, the K1 and K2 serotypes, which exhibit elevated levels of virulence in comparison with other serotypes, have been linked to more than 70% of known cases of hvKP infection worldwide [27]. Multilocus sequence types reveal greater sequence-type diversity in the K2 serotype compared with the K1 serotype, with K1 predominantly associated with ST23 and K2 linked to ST25, ST86, ST375, and ST380 [28,29]. There are differences in the distribution of serotypes across different geographic regions. In Asia, the most common serotype is K1 whereas in North America and Europe, K2 is more prevalent [30,31]. For MDR-hvKp convergent strains, the most prevalent serotypes in China are K64 and K47, which are related to ST11 [32]. Recombinations involving the *cps* synthesis locus are considered a major contributor to genetic diversification [33]. The subclone ST11-KL47 has been gradually replaced by ST11-KL64 as the dominant variant, with ST11-KL64 evolving enhanced pathogenicity, which has resulted in a significant increase in 30-day mortality rates [34]. The successful prevalence of this subclone is associated with enhanced antioxidant capacity, which in turn enhances the survival of ST11-KL64 in macrophages [35].

The capsular structure varies considerably between different serotypes, resulting in different degrees of virulence. Zhang et al. showed that the capsule type or composition has a direct influence on *K. pneumoniae* virulence traits by switching the expression of distinct *cps* gene clusters. In isogenic capsule mutant strains, K23 *K. pneumoniae* bacteria were cleared more rapidly than K3 *K. pneumoniae*, indicating that the capsule type also influences the host’s ability to kill bacteria [9].

Studies have indicated that K1 and K2 *K. pneumoniae* demonstrate greater virulence compared with strains of other serotypes [36,37], which is partly attributable to their greater resistance to phagocytosis and intracellular killing by immune cells, such as alveolar macrophages; additionally, this enhanced resistance is independent of whether they are hypercapsular strains [38]. Regarding liver abscesses induced by *K. pneumoniae*, the K1 and K2 serotypes have a greater impact on virulence levels as compared with the *magA* and *rmpA* genes [39]. Consequently, serotypes are considered potential virulence factors, maybe owing to their distinct components that provide a survival advantage. The capsules of K1 and K2 strains lack the mannose present in low-virulence strains. The presence of mannose facilitates recognition of capsules by macrophage lectin receptors and subsequently triggers phagocytosis. This absence inhibits efficient lectinophagocytosis and subsequent proinflammatory signals [40]. Sialic acid, found in K1 and K2 CPS, can imitate the sialic acid generally produced by host cells, facilitating evasion of the host immunological response or neutrophil repulsion by binding to Sia-binding immunoglobulin-like lectin-9, a major histocompatibility class I receptor located on neutrophils [38,41]. Furthermore, fructose-1,6-bisphosphate aldolase, a protein exposed on the bacterial surface, has been detected in K1 hvKP. Its higher levels of production can protect bacteria from being phagocytosed and killed by neutrophils in high-glucose environments [42].

Not all strains within the same serotype exhibit the same level of high virulence. K1/K2 serotype strains that do not have increased viscosity, and extra virulence factors usually do not show a hypervirulent phenotype. A study of K47 *K. pneumoniae* using a mouse peritoneal infection model found both high-virulence and low-virulence phenotypes, which could be attributed to sequence variations in the *cps* locus [9]. However, the accurate identification of hvKP serotypes at an early stage has important clinical consequences, including the detection of hidden abscesses for source control and surveillance of endophthalmitis [43].

## Synthesis and regulation of CPS

### Genes involved in CPS synthesis

**Figure 1.**
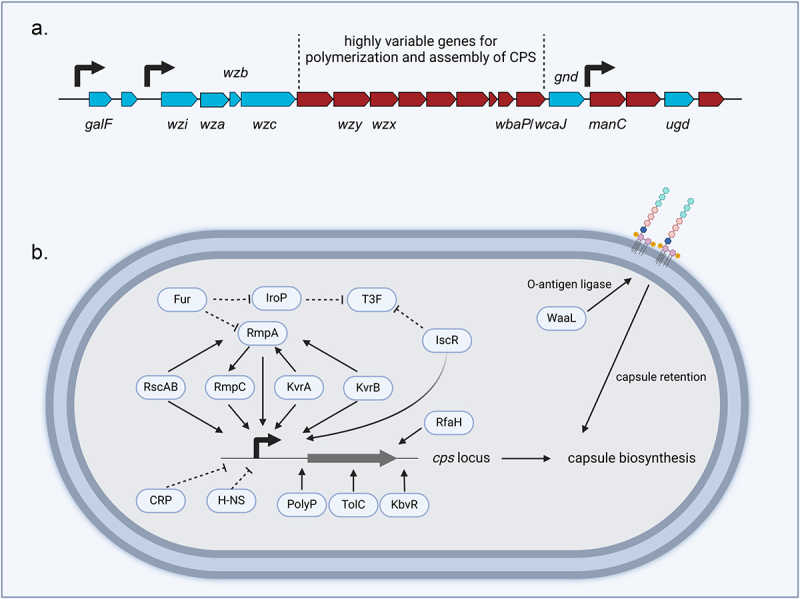
A. Schematic of CPS biosynthesis gene cluster. The right-turn arrows indicate the three known upstream promoters of the CPS synthesis region, blue genes represent those that are highly conserved across different K types, and red genes denote those that are highly variable. B. Regulatory factors of CPS in *Klebsiella pneumoniae*. Solid arrows denote promotion, and dashed arrows indicate repression. Different transcriptional regulators affect capsule biosynthesis via different mechanisms (figures created with BioRender.com).

The genes responsible for CPS synthesis in *K. pneumoniae* are located in the chromosomal *cps* synthesis region. The *cps* gene cluster spans approximately 21–30 kb and includes over 20 genes, from *galF* to *ugd* involved in capsule production (Figure 1). Three identified promoters are present in the *cps* locus, and these are situated upstream of *galF*, *wzi*, and *manC*. The 5' end of the *cps* region is a highly conserved cluster composed of *galF*, *cpsACP*, *wzi*, *wza*, *wzb*, and *wzc*, with > 50% nucleotide sequence similarity, which is involved in CPS translocation, transportation, and processing of surface proteins in bacteria. The 3' end contains the highly conserved genes *gnd* (encoding glucose-6-phosphate dehydrogenase) and *ugd* (encoding uridine diphosphate-glucose dehydrogenase). The highly variable middle region comprises specific genes encoding proteins that are essential for the polymerization and assembly of specific CPS subunits. This region typically contains genes encoding glycosyl transferases (GTs), translocases, polymerases, and modifying enzymes (such as acetyltransferases and pyruvoyltransferases), which vary among different capsule types. Each structural gene has a distinct impact on the virulence level [44].

*K. pneumoniae* synthesizes its CPS via the Wzx/Wzy-dependent mechanism, which involves both biosynthesis and export mechanisms. In the cytoplasm, nucleotide sugar precursors corresponding to a specific serotype are synthesized and assembled into repeating units on the lipid carrier undecaprenyl-pyrophosphate (Und-PP), facilitated by sugar-specific GTs [19]. The glycosylation of the repeating unit is initiated by *wbaP* (which links galactose to Und-PP) or *wcaJ* (which links glucose to Und-PP). Research has shown that *K. pneumoniae* is capable of having either WbaP or WcaJ, but not both simultaneously [44]. The protein encoded by *wbaP*/*wcaJ* mediates the first step of capsule biosynthesis, and its absence impairs capsule synthesis [45]. Some *wbaP* mutants can increase pathogenicity in urinary tract infection by promoting biofilm formation and invasion of the bladder epithelial cells [46]. Inactivation of the *wcaJ* gene decreases virulence and results in increased susceptibility to uptake by macrophages [47]. The flippase encoded by *wzx* flips the repeating unit to the periplasmic side of the inner membrane, and copolymerase encoded by *wzy* promotes polymerization of the repeating unit via a catch-and-release mechanism [48]. Subsequently, proteins encoded by *wza* (an outer membrane translocon) and *wzc* (a tyrosine autokinase) form a translocation complex that is involved in assembling the CPS and transporting it from the periplasm to the bacterial surface [49]. A cognate phosphatase of Wzc, Wzb binds to the catalytic domain and subsequently dephosphorylates Wzc [50]. This cycle of autophosphorylation and dephosphorylation is crucial for the polymerization and export of CPS via the Wzc-Wza complex. Truncation of *wzc* may alter the length of CPS or the number of repeating units, which results in reduced virulence [9]. Niu et al. showed that the lack of *wza* impairs CPS transport, leading to decreased expression of *wzb*, *wzc*, and *wzi*; this prevents the excessive accumulation of intracellular polysaccharide and ultimately results in capsule deficiency [50].

Inhibition of the Wzy-dependent pathway interrupts the synthesis of cell wall peptidoglycan and typically results in defects in cell shape defects if the cell survives. The *wzy*, *wza*, and *wcaJ* mutants all display a reduction in capsule biosynthesis, and the *wzy* and *wza* mutants also present substantial defects in cell envelope stability. This problem is presumably caused by the sequestration of undecaprenyl phosphate, as indicated by restoration of this phenotype via the inactivation of *wcaJ* [51].

Finally, CPS is anchored on a beta-barrel outer membrane protein encoded by *wzi*, which is not considered necessary for capsule biosynthesis but plays a vital function in capsule surface attachment to aid in capsule formation, such that mutants lacking *wzi* have in a capsule-deficient phenotype [20]. *wzy* is related to the polymerization of CPS. In 2004, chromosomal mucoviscosity-associated gene A (*magA*) was discovered as *wzy* located in the K1 locus, which has an important role in pyogenic liver abscess [52,53]. *magA* may serve as a virulence factor that is unique to K1 *K. pneumoniae* given that *magA* knockout strains lose their hypermucoviscous phenotype and resistance to lysis by the complement system [54].

## Genes involved in CPS regulation

### Rcs phosphorelay system

The Rcs phosphorelay system is a two-component signal transduction system that was initially discovered in 1985 as a positive regulator of colanic acid production in *Escherichia coli* [55]. This system comprises outer membrane lipoprotein RcsF, transmembrane sensor kinase RcsC, transmembrane protein RcsD, response regulator RcsB, and auxiliary protein RcsA [56]. Soon thereafter, it was revealed that this regulatory mechanism in *E. coli* also applies to *K. pneumoniae* [57].

The outer membrane lipoprotein RcsF serves as an intermediary between environmental stimuli and the Rcs phosphorelay system by transmitting signals caused by membrane stress or other elements to RcsC, ultimately triggering activation of the Rcs system [58]. The RcsC protein acts as a transmembrane sensor kinase that can autophosphorylate upon sensing extracytoplasmic stimuli. The phosphate signal is then relayed through RcsD to RcsB. RcsB may form either homodimers or heterodimers with auxiliary proteins such as RcsA, BglJ, GadE, and RmpA, among several others. Phosphorylated RcsB forms heterodimers with RcsA and binds to DNA sequences known as RcsAB boxes, thereby promoting the transcription of capsular synthesis genes [59]. Past research indicates that RcsAB may positively regulate the transcription of *galF* by binding to its promoter region, in this way influencing CPS synthesis and virulence levels [60].

Notably, the function of RcsA depends on phosphorylated RcsB whereas auxiliary proteins like MatA and GadE can operate regardless of whether RcsB is phosphorylated [61]. The genes involved in CPS synthesis and controlled by *rcsA* are limited in comparison with those that are controlled by *rcsB* in *K. pneumoniae* NTUH-K2044, and *rcsB* mutants exhibit reduced CPS expression and lower virulence levels (Figure 1) [60,62].

### Rmp regulators

RmpA and RmpA2 are essential regulatory factors that have a crucial role in the hypermucoviscous phenotype. In one study, a transposon library of mutations affecting CPS and hypermucoviscosity was screened, revealing that the production of CPS and the hypermucoviscous phenotype are closely linked [63].

Initially identified in 1989, RmpA possesses a LuxR-type DNA-binding domain, with its N-terminal region being specific to *Klebsiella* species [64]. The correlation between *rmpA*/*rmpA2*, hypermucoviscosity and hypervirulence is very strong, making it a proposed biomarker for detecting potential hvKP strains [65]. The protein encoded by *rmpA2* has a longer sequence than that encoded by *rmpA*, hence the designation *rmpA2* [66]. A comparison of the *rmpA* and *rmpA2* coding sequences reveals that these share approximately 80% homology.

Contrary to *rcsA* and *rcsB*, which are found on the chromosome, *rmpA* and *rmpA2* can be encoded by either plasmids or chromosomes. Chromosomal *rmpA* (*c-rmpA*) is located within the integrative conjugative element whereas plasmid-borne *rmpA* (*p-rmpA*) is more common. *rmpA2*, *rmpA*, and *rcsA* also share some homology [66]. One study reported that hvKP NTUH-K2044 contains both *p-rmpA* and *c-rmpA*, but only *p-rmpA* increases capsule production [67]. Another recent study indicated that the coexistence of c-rmpA, p-rmpA, and p-rmpA2 significantly enhanced virulence and the development of hepatic abscesses, compared with the coexistence of siderophores [68]. RmpA has been shown to promote the expression of *cps* by acting on three promoters that are located upstream of *galF*, *wzi*, and *manC* [67,69,70], and the *rmpA* knockout strain diminishes mucoviscosity and CPS synthesis [29].

Past research indicates that RmpA interacts with RcsB; it is hypothesized that RmpA can replace RcsA, thereby activating *cps* in the absence of RcsA [69]. Similar to RcsA, RmpA also relies on RcsB to activate CPS biosynthesis. Consistent with this theory, RcsB appears to be crucial for basal-level expression of the capsule from the *manC* promoter whereas RmpA can increase production of the capsule [70].

RmpA2 binds directly to the *cps* promoter to regulate capsule production [71]. In CG43, the *rmpA2* knockout strain loses its hypermucoviscous phenotype, but the production of CPS does not change significantly [69,71]. However, when *rmpA2* is overexpressed, the expression of CPS increases, suggesting that RmpA2 May only affect transcription under specific conditions.

RmpC is also predicted to encode a transcriptional regulator. The impact of *rmpC* knockout on CPS expression is similar to that of *rmpA* knockout; however, the *rmpC* knockout strain retains its hypermucoviscous phenotype. The knockout of *rmpC* distinctly separates the hypermucoviscous phenotype from the hypercapsular phenotype for the first time, suggesting that hypermucoviscosity and CPS production are distinct phenotypic traits that should be considered separately [70]. It has also been determined that *rmpC* is coexpressed with *rmpA*, and *rmpC* expression may be driven by RmpA [70]. Owing to the *rmpC* knockout strain exhibiting anti-phagocytic abilities that are similar to those of the wild-type strain, retention of the hypermucoviscous phenotype may key to evading host cell recognition and phagocytosis, leading to diminished virulence reduction in the *rmpC* knockout strain compared with the *rmpA* knockout strain. Furthermore, another study explored the distinct roles of CPS and hypermucoviscosity in host-pathogen interactions. The results demonstrated that hypermucoviscosity impedes bacterial adherence to host cells, and CPS shields bacteria from serum-mediated killing. CPS production is essential for *K. pneumoniae* to exhibit the hypermucoviscous phenotype, although other regulatory factors also contribute to hypermucoviscous phenotype [63]. The close, yet not entirely overlapping, relationship between CPS and hypermucoviscosity warrants further investigation.

The *rmpD* mutant strain also loses its hypermucoviscous phenotype, but *cps* is transcribed and there is no significant change in CPS production. In the *rmpADC* knockout strain, only the complementation of *rmpD* is sufficient to restore the hypermucoviscous phenotype. Thus, *rmpD* is the first gene proven to be essential for the hypermucoviscous phenotype [72]. Mutations in *wcaJ* and *manC*, required for CPS synthesis, both result in a hypermucoviscous phenotype that cannot be reversed by the expression of *rmpD*, suggesting that CPS synthesis is required for the hypermucoviscous phenotype [72]. Studies have shown that RmpD can interact with Wzc, making the CPS chains longer and more uniformly distributed in length, thereby altering the hypermucoviscous phenotype in different bacterial species [73].

### Other regulators

The iron-responsive transcriptional regulator Fur attaches to the upstream region of the *rmpA* promoter and suppresses its expression [69]. In *fur* knockout strains, the transcription of *rcsA* is increased [74], suggesting that the effect of *fur* on the capsule may be indirectly mediated via *rmpA* and *rcsA*. When iron levels are rich, Fur indirectly inhibits CPS expression. Another iron-responsive transcriptional regulator, IscR, has been proven to repress type 3 fimbriae (T3F) and enhance the expression of CPS from the *galF* and *manC* promoters [75]. Fur alleviates this inhibition when iron levels are low. Thus, *K. pneumoniae* appears to have acquired a mechanism to guarantee that CPS production is maintained at a basal level, at minimum, regardless of iron availability, and that permits changes in capsule production under extreme iron concentrations (Figure 1) [76]. A recent study described IroP, a novel regulator on the hvKP virulence plasmid, which inhibits T3F, thereby impeding biofilm formation and cell adhesion and potentially suppressing intestinal colonization. Furthermore, the presence of iron represses IroP expression via Fur. Acquiring this genetic switch allows for the synchronous inverse regulation of T3F and a hypermucoviscous capsule, alternating between a low mucoviscous capsule with a high T3F phenotype and a hypermucoviscous capsule with a low T3F phenotype, which facilitates adjustment to dynamic environments by modifying the ability to adhere to diverse surfaces [77].

KvrA and KvrB influence the virulence of K1 or K2 hvKP by activating the expression of *cps*, a mechanism that is not present in cKP. KvrA can affect *cps* expression via the *galF* and *manC* promoter regions, regardless of its impact on *rmpA*. This is in contrast to KvrB, whose influence on capsule gene expression relies solely on its interaction with the *rmpA* promoter [76,78]. The regulation of KrvA appears to be strongly influenced by the de-repression of histone-like nucleoid-structuring protein transcriptional silencing. The absence of *hns* depresses the production of CPS and downregulates type III pili [79].

cAMP receptor protein (CRP) can directly attach to the promoter regions of *wzi* and *manC*, decreasing the expression of three *cps* promoters and inhibiting CPS expression by regulating cAMP-dependent carbon catabolite repression. Knockout of *crp* increases viscosity, leading to greater glucuronic acid production [80,81]. CRP may also directly attach to the promoter region of *frwC*, which encodes the fructose-specific enzyme IIC, to regulate its expression. The deletion of *frwC* can promote *magA* transcription, thereby increasing CPS synthesis and biofilm formation [82].

Inorganic polyphosphate (polyP) is universally present in all living organisms and has a crucial function in promoting antibiotic resistance and enhancing virulence. PPK1 is the major enzyme involved in polyP synthesis. *ppk1* knockout strains exhibit reduced expression of the proteins involved in CPS formation and significantly decreased virulence levels, highlighting the critical role of polyP in hvKP [83]. TolC, an outer membrane pore protein, is necessary for the functioning of tripartite transport systems including ABC, major facilitator, and RND family transporters, which are associated with various types of antimicrobial resistance. Recent research has revealed that *tolC* knockout strains exhibit diminished CPS production, increased susceptibility to complement-mediated lysis, and lower expression levels of *cps* genes such as *galF*, *manC*, and *wza*. The mechanisms involved in the relationship between TolC and CPS still require further investigation. However, one potential mechanism is the inactivation of TolC-dependent transporters, leading to the intracellular accumulation of metabolites that are transporter substrates, which activates adaptive responses via feedback mechanisms to diminish CPS formation [84]. The *Klebsiella* biofilm and virulence regulator KbvR may influence the expression of *magA* or other regulators to influence capsule production. *kbvR* knockout strains exhibit reduced CPS, which decreases the resistance to innate immune defences and reduces virulence levels [85].

Finally, CPS can be maintained via interactions with other surface carbohydrate polymers, particularly the O antigen of the LPS molecule. The *waaL* deletion mutant lacks the ligase required for connecting the O antigen to the LPS core, leading to a significant decrease in capsule retention, which is related to greater susceptibility to human serum killing and lower virulence [86].

## Pathogenic mechanisms of CPS

**Figure 2.**
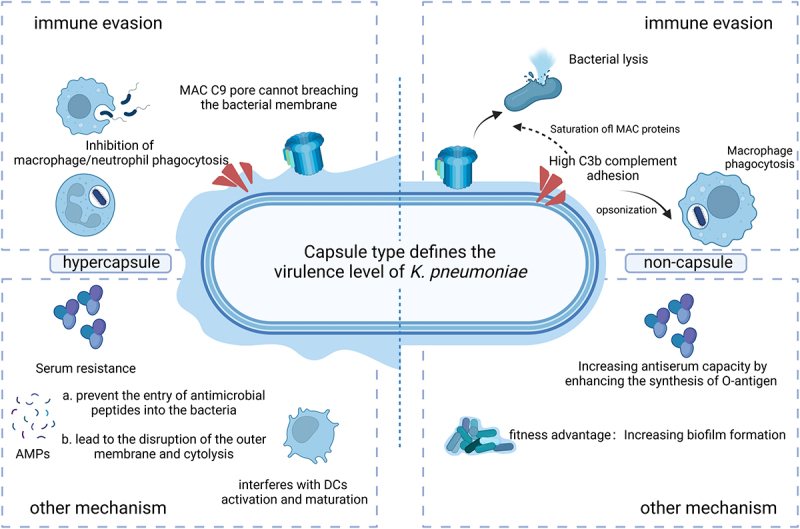
Pathogenic mechanism of *Klebsiella pneumoniae*. This schematic diagram illustrates interactions between the *K. pneumoniae* capsule and the host innate immune system, illustrating various mechanisms via which *K. pneumoniae* enhances its virulence and evades the immune response (figures created with BioRender.com).

The innate immune system acts as the initial barrier against bacterial invasion. Innate immune defences against *K. pneumoniae* infection, including phagocytosis by immune cells, complement-mediated killing, and antimicrobial peptides (AMPs) derived from the host, are the host’s principal strategies to clear infection (Figure 2). CPS can promote the survival of *K. pneumoniae* during infection and protect the pathogen, mainly by resisting complement-mediated lysis and avoiding recognition and targeted clearance by host immune cells, versus aggressively suppressing the immune response [87]. Diversity in the composition of CPS among different *K. pneumoniae* strains is a crucial factor in determining their ability to evade the immune system.

### Interaction between *Klebsiella pneumoniae* and the complement system

As an essential component of the host innate immune system, the complement system aids in the defences against pathogenic infection via two primary mechanisms. The first is through assembly of the membrane attack complex (MAC), which can lead to creation of pores in the gram-negative bacterial membrane and cause lysis of the bacteria. The second mechanism is C3b-mediated opsonophagocytosis of the bacterial surface to augment phagocytic cell function [8].

The proteolytic cascade of the complement system can be initiated via three extensively studied pathways: the classical pathway, the lectin pathway, and the alternative pathway. Depleting alternative pathway factors in *vitro* leads to enhanced survival of *K. pneumoniae* in serum [88]. Activation of these pathways leads to the formation of C3 convertases (C4b2b), finally coordinating the opsonization and subsequent elimination of bacteria. Subsequently, C3 convertase clears C3 into C3b and initiates MAC assembly as well as bacterial lysis by forming C5 convertase. Additionally, the deposition of C3b on surfaces can amplify the complement response by triggering activation of the alternative pathway, C3 convertase [89]. C3b can bind to complement receptors found on phagocytes, thereby inducing phagocytosis. By directly interacting with mannose-binding lectins, CPS (which includes mannose or rhamnose) can activate the lectin pathway and subsequently promote the opsonization of encapsulated *K. pneumoniae* [8,90].

*K. pneumoniae* can inhibit MAC-mediated lysis by producing thick CPS, representing one of the primary mechanisms against the host’s complement defense [90]. A possible explanation for this mechanism is that CPS acts as a physical barrier, blocking the assembly and deposition of complement system components and inhibiting MAC C9 pores from attaching to the bacterial membrane [90]. Moreover, thick CPS (rather than its composition) may impede the binding of other complement-activating subsurface structures, including LPS, outer membrane protein K36, and lectin-activating polysaccharide motifs [8,90]. In line with these findings, non-capsular mutants have greater amounts of C3 deposition and are more vulnerable to serum killing than strains with normal capsules. Capsular types K1, K10, and K16 have the ability to conceal their LPS. However, other capsular types like K2 lack this capability, thereby attenuating toll-like receptor 4 (TLR4) signaling in macrophages and leading to a reduction in mature cytokine production [54]. HvKp strains can change the composition of their CPS, bypassing identification via the lectin pathway [8,90].

A recent study reported a contradictory phenomenon in that *wcaJ*-inactivated strains displayed both elevated C3b complement deposition and increased resistance to serum killing; this may reflect failed complement activation [47]. Another study confirmed this phenomenon; the *wcaJ* mutation resulted in limited CPS biosynthesis while simultaneously improving serum resistance, strengthening bacterial fitness in the bloodstream; however, the high complement deposition also increased susceptibility to opsonophagocytosis. Increased bloodstream fitness and limited tissue pathogenicity may facilitate bacteria’s long-term survival in the host. A possible mechanism involved is that enhanced binding of complement components could result in the saturation of initial MAC proteins, which might cause steric hindrance that hampers the complete assembly of the sizable multi-protein C9 complex β-barrel structured pore [91,92]. Non-capsulated *K. pneumoniae* exhibits a fitness advantage by enhancing biofilm formation and antiserum capacity. However, it has shortcomings in terms of spreading, mortality, and resistance to phagocytosis. This may be attributed to the fact that disrupting CPS synthesis promotes O-antigen synthesis and eventually enhances serum resistance. The exact mechanisms need to be further investigated [93].

Clinical studies have demonstrated that genes related to CPS synthesis are involved in CPS-mediated complement resistance [94–97]. For instance, the transcription antiterminator RfaH, which is essential for *K. pneumoniae* fitness during lung infection [96], enhances CPS transcription. Inactivation of *rfaH* leads to diminished CPS synthesis, enhanced binding of C3b and MAC protein complexes, and diminished serum resistance [95]. This evidence suggests that thick CPS or the capacity to dynamically modify the CPS structure enables *K. pneumoniae* to elude detection by the immune system, which reflects the complexity of its immune evasion strategies [98].

### Interaction between *Klebsiella pneumoniae* and innate immune cells

In addition to the complement system, successful colonization of *K. pneumoniae* involves multitudes of interactions with various immune cells, such as macrophages and neutrophils, all of which function to eliminate invading pathogens. A thick capsule inhibits *K. pneumoniae* attachment to and engulfment by phagocytic cells.

Macrophages are the initial phagocytes involved in the containment of *K. pneumoniae* during an infection, such as lung infection [99,100]. Macrophages sense and initiate the appropriate mediators to stimulate the immune response against *K. pneumoniae*. For example, the CPS of KN2 carbapenem-resistant *K. pneumoniae* can activate macrophages to secrete cytokines including tumor necrosis factor-α and IL-6, which participate in the innate immune response [101]. *K. pneumoniae* can successfully infect the host by evading detection and phagocytosis by macrophages as well as by resisting intracellular killing after phagocytosis. The liver is a crucial organ for ensnaring invading pathogens involved in bloodstream infections [102,103]. Kupffer cells, which are liver-resident macrophages, constitute about 90% of all resident macrophages in the body and are the primary phagocytes in the sinusoids with a crucial role in the liver’s anti-infection function [104]. The ability of Kupffer cells to capture *K. pneumoniae* is determined by the bacterial capsule type, with high-virulence capsule type enhancing bacterial survival and virulence by evading capture and killing in the liver [9]. Hypercapsular mutants, resulting from a single nucleotide polymorphism in *wzc*, enhance the resistance to phagocytosis and the ability to spread, leading to increased mortality owing to bacterial infection. However, these mutants are incapable of forming biofilms or efficiently invading bladder epithelial cells, thereby limiting the severity of infection to some extent [105]. In contrast, capsule-deficient *K. pneumoniae* has shown greater adaptability in urinary tract infection in mice by more readily forming biofilms and invading bladder epithelial cells, thereby leading to persistent infection. Moreover, these bacteria cannot be effectively eradicated, even if they are antibiotic sensitive in *vitro* [46].

Mouse infection models have demonstrated that neutrophils are crucial for protecting against hvKP [106]. Research has shown that K1 and K2 strains exhibit notably higher resistance to phagocytosis and intracellular killing by neutrophils in comparison with non-K1 or -K2 strains [107]. Other investigations have corroborated these results and further demonstrated that escape of neutrophil-mediated killing is mostly accomplished by evading phagocytosis rather than inhibiting extracellular neutrophil extracellular trap (NET) formation [36]. An isogenic non-capsular mutant of K1 was found to be incapable of escaping neutrophil-mediated phagocytosis, suggesting that CPS partially facilitates immune evasion in hvKP [108]. Recent studies have shown that hypermucoviscosity and excessive production of CPS are two separable traits [72]. Identification of clinical strains that exhibit increased capsule production without the hypermucoviscous phenotype has confirmed that hypermucoviscosity mediated by *rmpA/A2*, rather than CPS, endows *K. pneumoniae* with the ability to avoid binding and engulfment by neutrophils.

CPS also interferes with dendritic cell (DC) activation and maturation, which can result in the impaired function of immature DCs during *K. pneumoniae* antigen presentation, with reduced expression of Th1 cytokines and TLR4. Natural killer cells are also reduced as a result of this impairment, in this way blocking their migration to the infection site [109].

### Other pathogenic mechanisms of *Klebsiella pneumoniae*

Antimicrobial peptides (AMPs) are naturally occurring small-molecule peptides, which are an essential part of the innate immune defence system and possess a wide range of inhibitory effects against bacteria [110]. Studies of *K. pneumoniae* capsules and AMPs have yielded different conclusions. Several investigations have demonstrated that the capsule can provide protection against AMPs [110]. Exogenous addition of CPS can bind antimicrobial peptides, including polymyxin, thereby preventing entry of AMPs into the bacterium and reducing the killing effect of polymyxin [111]. AMPs in sublethal quantities in the airway induce the expression of *cps*, which subsequently shields bacteria from the effects of AMPs [112].

As a type of AMP, polymyxins are often used as a last resort in clinical applications. However, a recent study suggested that the presence of capsules increases the susceptibility of *K. pneumoniae* to polymyxin. This idea has also been proven in numerous studies; for example, *wcaJ* or *wzc* loss-of-function mutations are more resistant to polymyxins [92,113,114]. This may be owing to the capsule enhancing the concentrations of AMPs attached to the bacterial outer membrane by binding to LPSs and phospholipids, thereby disrupting the outer membrane, inducing cytolysis, and enhancing the killing effect of AMPs [115,116]. In high alcohol-producing *K. pneumoniae*, glucose enhances drug resistance to polymyxins by repressing the expression of *crp*, which promotes CPS synthesis [117]. Therefore, the role and exact mechanisms of capsules in bacterial antibiotic resistance remain to be clarified.

By preventing respiratory pathway epithelial cells that express TLRs from secreting IL-8, CPS reduces inflammation by blocking any downstream immune response to IL-8 [13]. Furthermore, the capsule serve as a general protective mechanism against T6SS attacks [118] and is involved in the colonization of *K. pneumoniae* in the oropharynx and lower gastrointestinal tract [119].

## Conclusions and perspective

HvKP can cause severe community-acquired infections that have high pathogenicity and a high mortality rate. In recent years, *K. pneumoniae* strains with high virulence and antibiotic resistance, especially resistance to carbapenems, have spread widely worldwide, posing a serious threat and challenge to public health. CPS is an important virulence determinant that aids in evading the host innate immune system and promotes survival during *K. pneumoniae* infection. The numerous capsule regulatory factors also indicate the crucial role of the capsule in bacterial virulence levels. Further in-depth research is needed on the pathogenicity and immune regulatory capabilities of CPS.

CPS is a potential therapeutic target in *K. pneumoniae* owing to its substantial immunomodulatory characteristics, and capsule-targeting antibodies have been shown to increase killing of *K. pneumoniae* [120]. A biconjugate vaccine composed of K1 and K2 serotype CPS could protect mice from severe pulmonary infection caused by hvKP [121,122]. Moreover, an inactivated whole-cell vaccine against *K. pneumoniae* could protect the host from lethal bloodstream infection owing to hvKP [9]. A recent study comparing two bioconjugate vaccines against the K2 serotype and the O1 O-antigen showed that capsule-targeted vaccines may be more effective than O-antigen vaccines because the presence of *K. pneumoniae* capsules prevents the binding and functioning of O1-antibodies [123]. The mechanisms of protective immunity induced by *K. pneumoniae* vaccines, as well as the development and optimization of such vaccines, require further exploration. The numerous capsular serotypes of *K. pneumoniae* present a considerable challenge to achieving broad vaccine coverage. Therefore, detecting clinically prevalent serotypes to guide vaccine development is crucial.

Currently, scientists are investigating strategies including combination therapies that combine antibodies and antibiotics to enhance treatment effectiveness. For instance, Pennini et al. found human monoclonal antibodies that are specific to O-antigen in combination with antibiotics prevented death in mice infected with MDR *K. pneumoniae* [124]. K1 and K2 CPS-conjugated vaccines generated by CPS depolymerases identified in phages are also promising candidates for use in vaccines against *K. pneumoniae* infection [125]. It is crucial to prioritize the translation of these early-stage results into clinical practice, particularly as the prevalence of MDR-hvKP continues to rise.

## Data Availability

Data sharing is not applicable to this article as no datasets were generated or analyzed during the current study.

## Author statement

All authors have read and approved the final work.

**Li Xu**: Writing – original draft, Investigation, Conceptualization, Project administration.

**Jiayang Li**: Writing – original draft, Investigation, Conceptualization, Project administration.

**Wenqi Wu**: Investigation, Project administration.

**Xiuwen Wu**: Writing – review & editing, Funding acquisition, Supervision, Resources.

**Jianan Ren**: Writing – review & editing, Funding acquisition, Supervision, Resources.
